# Enhancing cyberattack resiliency through the radiotherapy backup and recovery dashboard tool

**DOI:** 10.1002/acm2.70292

**Published:** 2025-10-22

**Authors:** Justin Pijanowski, Eric Nguyen, Yasin Abdulkadir, Justin Hink, Yevgeniy Vinogradskiy, James Lamb

**Affiliations:** ^1^ Department of Radiation Oncology University of California Los Angeles Los Angeles California USA; ^2^ Department of Radiation Oncology Thomas Jefferson University Hospital Philadelphia Pennsylvania USA

**Keywords:** cyberattack resiliency, radiotherapy backup software, radiotherapy data restoration

## Abstract

**Purpose:**

Radiation Oncology departments impacted by recent cyberattacks were unable to access data backups or their Record and Verify (R&V) system and therefore faced challenges to resume patient treatments in a timely manner. We present a novel software tool that backs‐up critical radiotherapy treatment information and displays essential information for on‐treatment patients in an intuitive and accessible dashboard allowing clinics to continue radiotherapy treatments. The purpose of this report is to describe implementation details, challenges, and share open‐source code to facilitate radiation oncology clinics’ efforts to develop tools to improve cyberattack resiliency.

**Methods:**

The Radiotherapy Backup and Recovery Dashboard Tool (RBRDT) performs daily backups of treatment information and relationships between DICOM‐RT objects from the R&V system and treatment planning system (TPS) to a Radiation Therapy Picture Archiving and Communication System (RT‐PACS). If the R&V system is inaccessible, the RBRDT accesses the backed‐up data in the RT‐PACS to create a dashboard containing critical treatment information for patients currently undergoing radiotherapy.

**Results:**

The RBRDT is successfully clinically implemented and generates backups to the RT‐PACS every 10 minutes. Since its implementation in May 2024, the RBRDT backed up over 80,000 RTRECORDS and moved 322 RTPLANS and 142 RTSTRUCTS to the RT‐PACS that were absent from this server caused by human error. The dashboard is generated nightly.

**Conclusions:**

The RBRDT fills a critical gap in existing approaches for radiotherapy treatment data backup and restoration. All relational data is transferred to the RT‐PACS which is accessible during a cyberattack, reducing the reliance on the R&V system. The RBRDT retrieves connected data elements saved in separate locations. In the event of a cyberattack, this data is returned in a dashboard to rapidly resume patient treatments.

## 1 INTRODUCTION

Radiation oncology is a medical discipline that relies heavily on computerized systems—including treatment planning, delivery, and automated checks to perform nearly all tasks, making departments particularly vulnerable to cyberattacks. Recently, ransomware attacks, a form of cyberattack have targeted Radiation Oncology departments throughout the country.^1^, ^2^, ^3^, ^4^, ^5^, ^6^, ^7^, ^8^ These attacks resulted in loss of access to systems critical to patient care, including Electronic Medical Record (EMR) systems, Record and Verify (R&V) systems, and secure communication systems. Consequences include delayed patient treatment, early treatment termination, and higher probability of errors when restarting treatment. Delays in resuming treatment are especially harmful in radiation therapy due to accelerated tumor growth if radiotherapy treatments are missed,^9^, ^10^, ^11^, ^12^, ^13^, ^14^, ^15^, ^16^ directly translating into worse clinical outcomes. Thus, it is vital to resume treatment safely and quickly following a ransomware attack.

Medical networks and Radiation Oncology departments impacted by ransomware attacks have backup systems for patient and treatment information. However, infrastructure‐level backups, e.g. of entire applications are at risk of themselves being infected with ransomware and may be quarantined per security policy. The University of Vermont Health Network describes^1^, ^2^ that immediately following the attack, all access to their EMR, R&V system, clinical systems, and secure communication systems were halted, preventing access to their backed‐up R&V server. Patient treatment plans beyond two days following the attack were inaccessible. Health Sciences North^4^ details the ransomware attack on their network prevented access to their R&V system, forcing them to manually reconstruct each patient record utilizing multiple sources of information. This process involves significant amounts of time, resources, and is prone to human error. Similar manual efforts were taken by other Radiation Oncology departments and healthcare institutions when targeted by ransomware attacks.^5^, ^7^, ^8^

In response, we developed a robust software application called the Radiotherapy Backup and Recovery Dashboard Tool (RBRDT) to mitigate disruptions from potential cyberattacks by providing streamlined backup and retrieval of critical information necessary for continued clinical operations. The RBRDT performs daily backups of treatment records from a Digital Imaging and Communication in Medicine (DICOM)‐compliant^17^ R&V system to a Radiation Therapy Picture Archiving and Communication System (RT‐PACS). The RBRDT accesses the RTPACS nightly to generate a dashboard of critical treatment information for patients currently undergoing radiotherapy, which can be used to rapidly resume treatments if the R&V system is inaccessible. This report describes implementation, challenges, and shares open‐source code to assist other clinics’ bolster their own cyberattack resiliency.

## 2 METHODS

The RBRDT was implemented for external beam radiation therapy (EBRT) only using the Python software package pynetdicom,^18^ a Python implementation of the DICOM networking protocol. Hosted on a virtual machine, it automatically runs every ten minutes, seven days a week using Windows Task Scheduler. Our R&V system is ARIA (Varian Medical Systems, California, United States) and the RT‐PACS is MIM (MIM Software Inc., Cleveland, OH), however the RBRDT is compatible with any DICOM‐compliant R&V and RT‐PACS system.

### 2.1 Network Configuration

**FIGURE 1:**
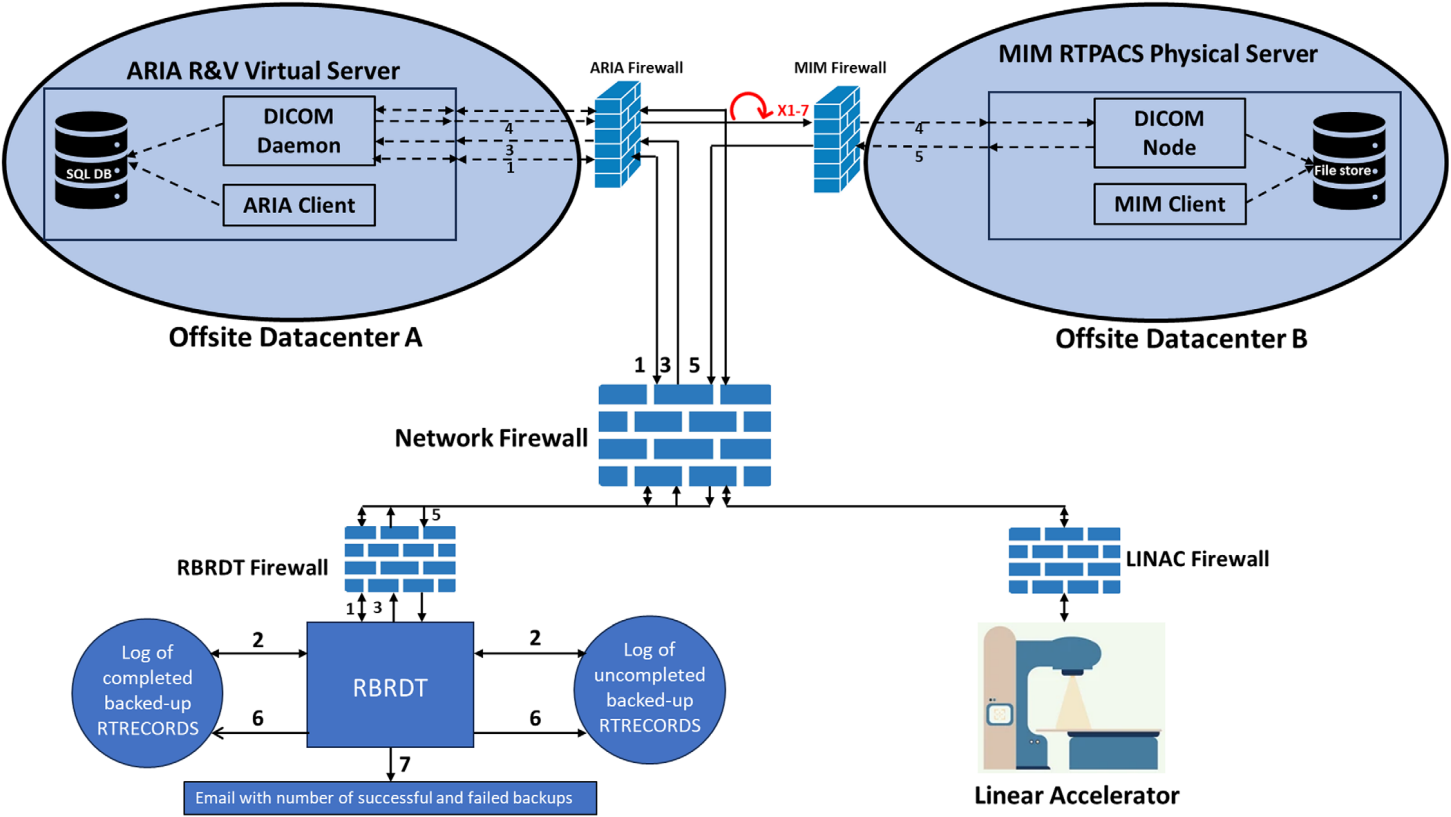
Diagram depicting our network architecture and the functions of the RBRDT to back‐up RTRECORDs to the RT‐PACS. 1): Query the R&V system and retrieve RTRECORDs. 2): Compare the returned RTRECORDs to the completed backups. 3): RBRDT initiates a C‐MOVE. 4): Pending backups are transferred from the R&V system to the RT‐PACS. Failed backups are repeated up to seven times. 5: Success or failure status for each backup is returned to the RBRDT. 6): The appropriate logs are updated according to the returned status. 7): Nightly email is generated detailing number of successful and unsuccessful backups.

The ARIA and MIM databases are hosted in separate offsite datacenters. Neither database is cloud‐based. ARIA operates on a virtual server, and MIM is hosted on a physical server, both within our institution's data centers. Our network configuration is displayed in Figure 1. In addition to being in a different datacenter to ARIA, the RT‐PACS is protected using firewall technology. A client connection to the RT‐PACS server through DICOM association requires the client IP address, port number, and Application Entity Title to be known by the server, thus preventing access to the RT‐PACS by any unauthorized clients. Because the two systems operate independently, each with their own servers, firewalls, and access controls, it is unlikely that a cyberattack on ARIA would simultaneously compromise MIM.

### 2.2 RT Record Backup

Figure 1 displays the backup process. After each fraction of EBRT, the linear accelerator (LINAC) sends the treatment details to the R&V system, which can be queried for and retrieved as a DICOM‐RT record (RTRECORD) object. Each morning, the RBRDT initializes a log file that stores the unique identifiers, called SOPInstanceUIDs (UIDs), of the successful RTRECORD back‐ups to the RT‐PACS for that day. The RBRDT sends a C‐FIND request to the R&V to query for all the day's RTRECORDs. This query returns a list of the RTRECORD UIDs that represent the completed treatments. RTRECORDS cannot be queried by treatment time, which would be preferable. Since the average EBRT appointment time is between 15 and 30 minutes, the backup frequency of 10 minutes was chosen to minimize potential loss of unbacked‐up data and avoid excessive strain on system resources from overly frequent backups.

To prevent redundant copying of backed‐up RTRECORDs, the returned RTRECORD UIDs are compared against the UIDs stored in the daily log file. For each unique RTRECORD, the RBRDT sends a C‐MOVE request to the R&V system to transfer this RTRECORD to the RT‐PACS. If the connection to the RT‐PACS from the R&V system fails, or the RTRECORD transfer fails, the RBRDT retries the backup up to seven times. If these attempts fail, the UID of the RTRECORD is added to a failed‐transfer log file. RTRECORD UIDs later backed up successfully will be removed from the failure log file. The RBRDT generates a nightly email to our clinical staff containing the number of successful backups for that day and details of failed backups. Our team manually transfers failed backups from the R&V system to the RT‐PACS. The content of these email notifications allows us to verify the RBRDT system status. A minimal number of backups during clinical operations suggests the RBRDT is non‐operational.

### 2.3 Hierarchical Search for Referenced Plan Objects

**FIGURE 2:**
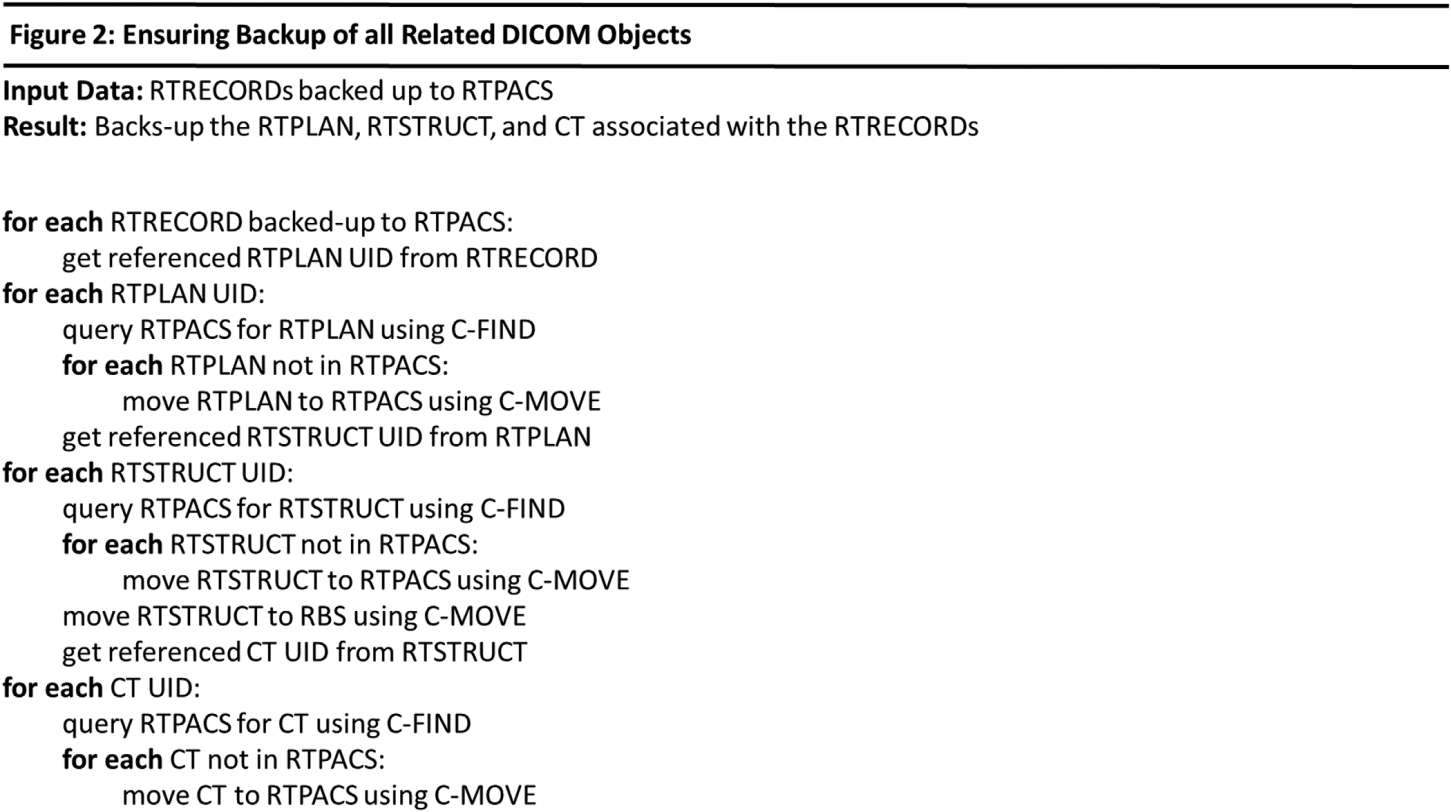
Ensuring Backup of all Related DICOM objects.

Treatment plan information (RTPLAN, RTSTRUCT, and CT Image) corresponding to each RTRECORD may be needed for patient care during a cyberattack. These objects should exist within the RT‐PACS since our planning workflow pushes plans to the RT‐PACS after plan approval. This manual archiving process is imperfect and therefore the RBRDT checks for existence of these objects and recovers them from the R&V and/or TPS if necessary. Our TPS (Eclipse, Siemens Healthineers, Forcheim, Germany) shares a common database with the R&V (ARIA), but the RBRDT can easily be configured to query a separate TPS. The RTRECORD, RTPLAN, RTSTRUCT, and CT Image are organized as a linked list data structure where each subsequent object is referenced by the one prior. Figure 2 shows how these objects are backed up in the RT‐PACS, which are performed each time the RBRDT runs.

**FIGURE 3:**
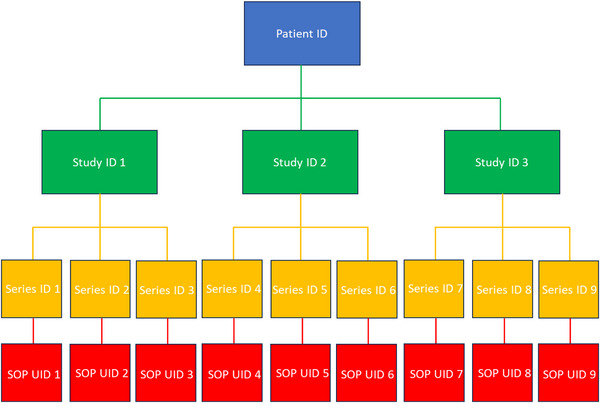
Figure detailing the hierarchical query implemented for compatibility with MIM RT‐PACS. Blue rectangles indicate the patient level, green indicates the study level, yellow indicates the series level, and red indicates the instance level. The UID of the previous level must be known to query at the next level.

Planning objects are referenced by RTRECORDs via their SOPInstanceUIDs. MIM RT‐PACS only supports hierarchical querying, which behaves like tree traversal, where access to a specific node (object) requires providing the UIDs for all levels above it. However, since these higher‐level UIDs (e.g., for Patient, Study, or Series) are not readily available, we implemented a breadth‐first search algorithm to identify the full path to the desired object and all necessary UIDs for the query. Figure 3 shows the DICOM hierarchical tree structure.

### 2.4 Current Patients Dashboard

If a ransomware attack renders the R&V system inaccessible, the RBRDT utilizes the backed‐up data in the RT‐PACS to return critical treatment information in a CSV file for patients currently receiving radiotherapy treatment. This information can be displayed in a human‐readable Excel dashboard. In the situation where Microsoft Office services are offline, the CSV file can be viewed in any text viewer. We define patients currently being treated as those who have not completed all planned fractions and whose most recent fraction was delivered within the past seven days. Our RT‐PACS server only supports hierarchical querying, thus we first query for all records based on the Study Date DICOM tag, indicating when the simulation CT was created. We then filter to obtain the needed RTRECORDs. We select the Study Date to be two months prior to the current date to include patients with long treatments. This query returns a list of UIDs for RTRECORDs within the date span. If multiple RTRECORDs are returned for a single patient, the RBRDT keeps the most recent RTRECORD. These RTRECORDs represent patients undergoing radiotherapy treatment.

**FIGURE 4:**
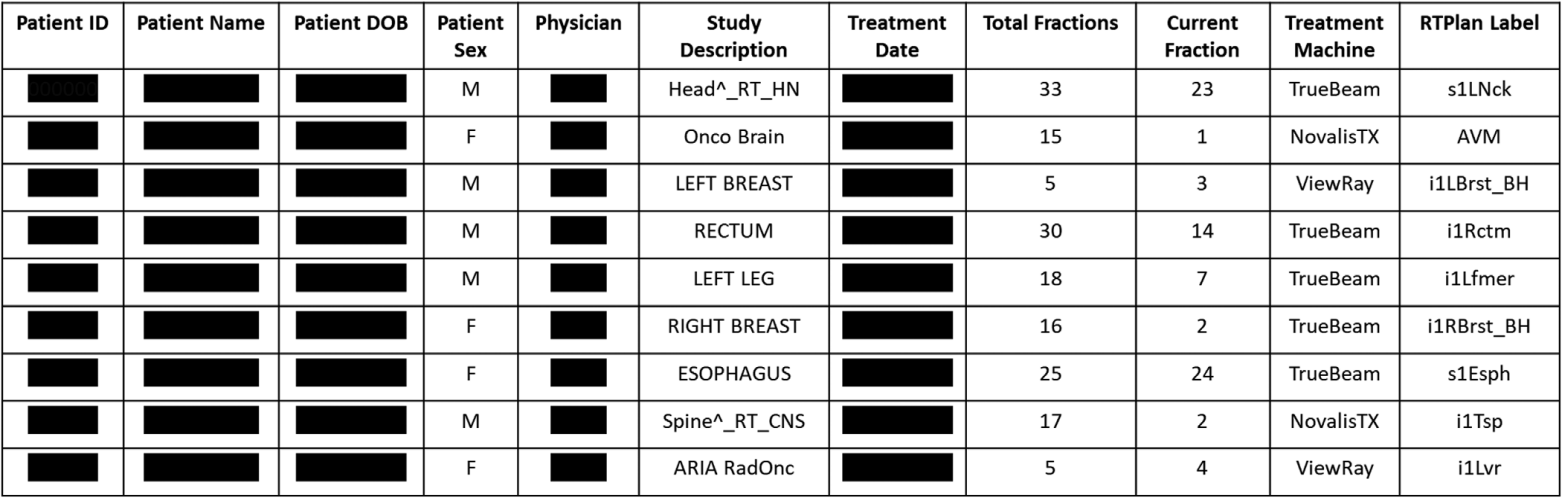
Excel dashboard containing critical information necessary to rapidly resume radiotherapy treatments.

Elements of the Excel dashboard, shown in Figure 4, include Patient Name, MRN, DOB, Physician of Record, the number of fractions planned and the current fraction number. In the dashboard, each line will reference one radiotherapy plan and contains the above information. If there are multiple plans per patient, these plans are grouped together, but listed as separate entries on individual lines. In the event where the R&V system is inaccessible, the Excel dashboard will be utilized to swiftly resume patient treatments.

## 3 RESULTS

**FIGURE 5:**
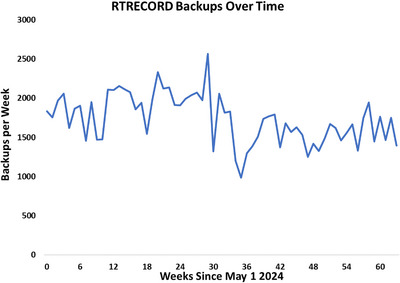
Line chart detailing the number of monthly RTRECORD backups since commissioning.

The RBRDT is fully implemented in our clinic and performs backups to the RT‐PACS every 10 minutes throughout the day. Numerical results of these backups are shown in Figure 5. To date, the RBRDT has backed‐up over 80,000 RTRECORDS, 322 RTPLANs, and 142 RTSTRUCTS to our RT‐PACS. These RTPLANs and RTSTRUCTS were missing from the RT‐PACS due to human error. The Excel dashboard is generated nightly by the RBRDT and contains the data for the actively treated patients.

## 4 DISCUSSION

The RBRDT was designed to aid Radiation Oncology clinics in resuming patient treatments efficiently and accurately following a cyberattack. Challenges overcome included algorithmically determining the patients currently being treated from the DICOM metadata and the implementation of hierarchical querying for DICOM objects from our RT‐PACS.

Zhang et al.^19^ implemented a ransomware‐focused backup system where treatment information was stored on a secure workstation, and a clean R&V system without patient data was installed on a second workstation. If the clinical R&V system was disabled, DICOM objects and EMR files from the secure workstation would be manually transferred to the clean R&V system. The vendor would reconfigure each treatment machine to use this secondary system. This approach is robust but time consuming and vendor dependent. Our methodology supports treatment using the LINAC's file mode with the RTPLAN object. This complementary approach is designed for rapid continuation of treatment until vendor support is obtained.

A limitation of our work is the RBRDT has not been implemented at outside institutions to increase the robustness of the RBRDT. Currently, the RBRDT is compatible with R&V systems and RT‐PACS that support DICOM query and retrieval. For example, radiation oncology clinics using the MOSAIQ R&V system (Elekta, Stockholm, Sweden) must access their relational database using structured query language (SQL) as MOSAIQ is not DICOM compliant for treatment records. The current version of the RBRDT is incompatible with MOSAIQ and other R&V systems that do not have a DICOM query‐retrieve interface.

While ARIA and MIM are not hosted in a cloud environment, a cloud‐based architecture could be implemented. The functionality of the RBRDT would be largely identical if ARIA or MIM were hosted in the cloud. Firewall modifications to allow the RBRDT to associate with cloud‐based servers would not be notably different from our current practices, as our institution adheres to a zero‐trust security framework where network access is tightly controlled, even internally.

Future work includes testing the RBRDT at an institution with an identical R&V system to our own. Secondly, the RBRDT will be modified to backup an R&V system that does not support DICOM query and retrieval, such as MOSAIQ. We are engaged in multi‐institutional cybersecurity simulation exercises designed to prepare radiation oncology clinics for cyberattacks. While a complete description is outside the scope of this manuscript, these comprehensive exercises will implement the functional RBRDT to aid in resuming patient treatments and allow us to improve features of the RBRDT. We plan to further develop the dashboard to include a graphical user interface, increasing user accessibility.

**FIGURE 6:**
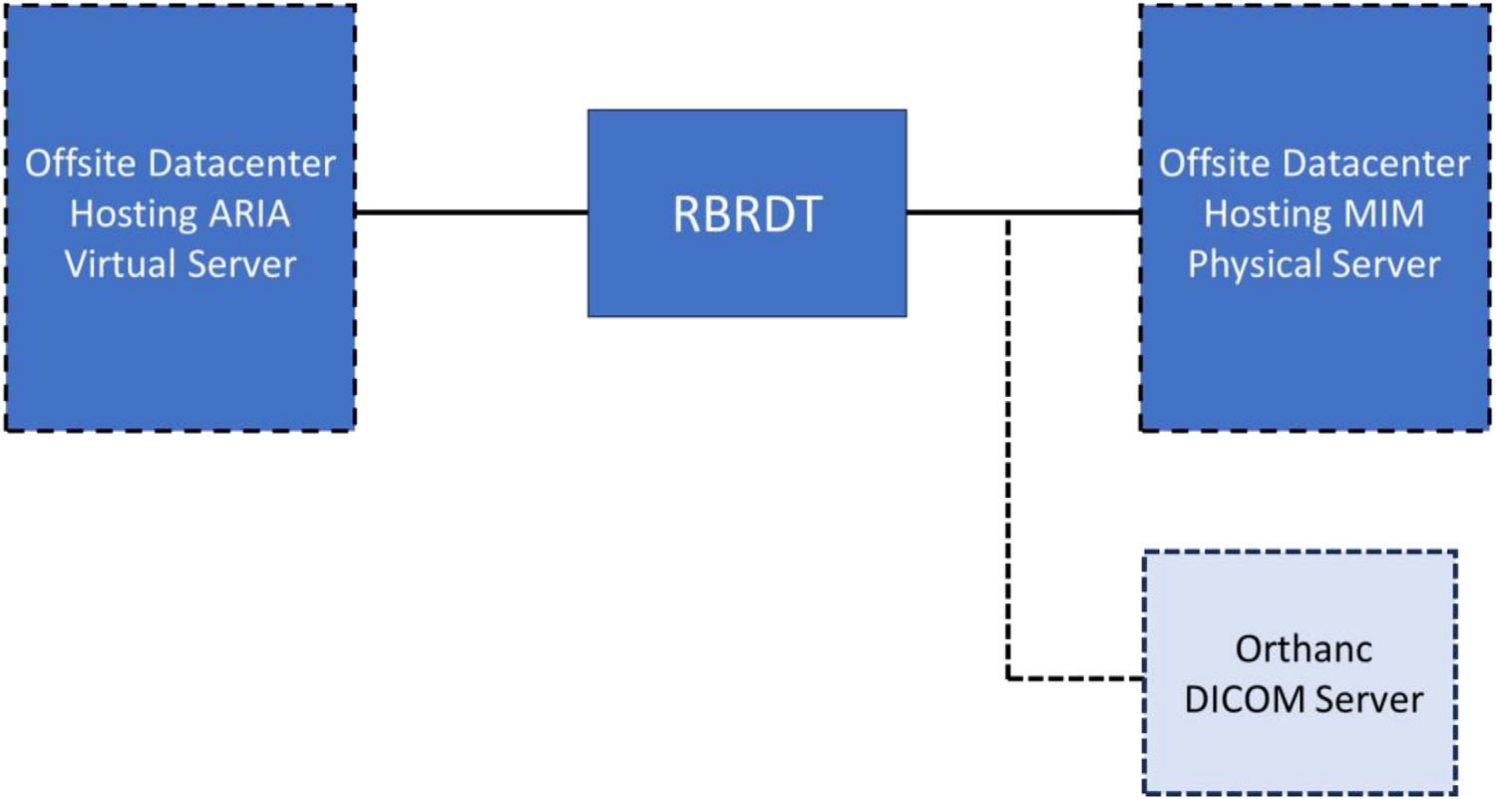
Optional configuration where a Orthanc DICOM server can serve as a siloed RT‐PACS.

Nelson et al.^20^ suggest establishing “siloes” that are exempt from network shutdowns to store relational data. While our current approach demonstrates the concept of the RBRDT, future work involves integrating these suggested siloes into our software to prevent the RBRDT from becoming infected in the event of a cyberattack. Currently, our DICOM data is backed‐up to our RT‐PACS in a datacenter. One implementation of a silo is to deploy a local RT‐PACS workstation in our clinic that allows offline DICOM query and retrieval from this RT‐PACS to eliminate reliance on the hospital network for these tasks. A second approach is shown in Figure 6. An Orthanc^21^ DICOM server can be deployed on a workstation to serve as a lightweight RT‐PACS. DICOM data can be backed‐up to this Orthanc server and reside locally on the workstation. To reduce time accessing the network, the Orthanc server will remain offline and only activate when the RBRDT is ready to transfer data.

## 5 CONCLUSION

Recent cyberattacks attacks on healthcare institutions demonstrated the vulnerability of networks to this form of attack. The Radiotherapy Backup and Recovery Dashboard Tool (RBRDT) provides Radiation Oncology departments with novel methods to enhance their cyberattack resiliency and swiftly resume patient treatments. The RBRDT source code has been released publicly^1^ under the MIT License to aid clinics in their efforts to improve resilience to potential cyberattacks.

## AUTHOR CONTRIBUTIONS

Yevgeniy Vinogradskiy and James Lamb developed the concept behind the research. Justin Pijanowski and Eric Nguyen engineered the operational design of the project and wrote the software for this design. Yasin Abdulkadir and Justin Hink supported in developing the software. Justin Pijanowski and Eric Nguyen composed and edited the manuscript under the support and guidance from Yevgeniy Vinogradskiy and James Lamb. Yasin Abdulkadir and Justin Hink provided edits to the manuscript.

## CONFLICT OF INTERESTS STATEMENT

The authors have no relevant conflicts of interest to disclose.

## Footnotes

^1^

https://gitlab.com/ucla_rosaml/jpijanowski/rbrdt‐refactored
